# Design of Low-Complexity Convolutional Neural Network Accelerator for Finger Vein Identification System

**DOI:** 10.3390/s23042184

**Published:** 2023-02-15

**Authors:** Robert Chen-Hao Chang, Chia-Yu Wang, Yen-Hsing Li, Cheng-Di Chiu

**Affiliations:** 1 Department of Electrical Engineering, National Chung Hsing University, Taichung 40227, Taiwan; 2 Department of Electrical Engineering, National Chi Nan University, Nantou 54561, Taiwan; 3 Department of Electrical and Computer Engineering, Cornell University, Ithaca, NY 14850, USA; 4 Neurosurgical Department and Spine Center, China Medical University Hospital, Taichung 404332, Taiwan

**Keywords:** finger vein, CNNs, batch normalization, SDUMLA-HMT, contrast limited adaptive histogram equalization (CLAHE)

## Abstract

In the biometric field, vein identification is a vital process that is constrained by the invisibility of veins as well as other unique features. Moreover, users generally do not wish to have their personal information uploaded to the cloud, so edge computing has become popular for the sake of protecting user privacy. In this paper, we propose a low-complexity and lightweight convolutional neural network (CNN) and we design intellectual property (IP) for shortening the inference time in finger vein recognition. This neural network system can operate independently in client mode. After fetching the user’s finger vein image via a near-infrared (NIR) camera mounted on an embedded system, vein features can be efficiently extracted by vein curving algorithms and user identification can be completed quickly. Better image quality and higher recognition accuracy can be obtained by combining several preprocessing techniques and the modified CNN. Experimental data were collected by the finger vein image capture equipment developed in our laboratory based on the specifications of similar products currently on the market. Extensive experiments demonstrated the practicality and robustness of the proposed finger vein identification system.

## 1. Introduction

Security verification technologies have advanced rapidly in modern society. For example, airport check-in systems have advanced from manual to automatic inspections. With the increasing focus on personal privacy, much attention has been paid to biometric verification, which utilizes human physiological characteristics, such as features of the sclera [1], fingerprints [2], veins [1,3,4], and face [5], for identity verification. All these technologies have three things in common. First, every person has their own biometrics (i.e., “uniqueness”). Second, these features do not change dramatically over time (i.e., “stability”). Lastly, users need not bring all their personal keycards with them, and these biometric features allow accurate identification and are hard to spoof or steal (i.e., “safety and portability”). With these three advantages, biometrics has become the main trend for personal identification in modern society.

Finger vein features used in human recognition methods, whether it is image processing of photographic devices or finger vein feature recognition algorithms, have been implemented in various applications that require security, such as ATMs and security doors [6,7]. The finger vein identification system recognizes the structure of the visible blood vessel pattern in the finger, which can only be irradiated with near-infrared (NIR) light wavelengths. Because finger vein patterns are located under the skin, they are neither changeable over time nor easily affected by skin surface changes, such as cuts, abrasions, and surface stains, or by interference noise [8].

In recent years, convolutional neural networks (CNN) have been widely used in many feature extraction and classification tasks. The CNN uses convolution kernels of different size to obtain the detailed features of images, including for finger vein recognition. Traditional CNN for vein recognition had been known for having heavyweight architectures [9], which required large amounts of computational resources and data for training and inference. This led to the development of lightweight CNNs that are more efficient in terms of computational resources and memory usage. Several recent works have proposed lightweight CNN architectures to improve the performance of the recognition task while reducing the computational cost. One such system used a lightweight CNN with triplet loss function, composed of stem blocks for extracting coarse features of the images and stage blocks for extracting detailed features [10]. Another system proposed a unified CNN which achieved high performance on both finger recognition and anti-spoofing tasks while making their neural network compatible [11]. It is interesting to note that there have been several recent papers proposing lightweight CNN architectures for finger vein recognition tasks and this topic has been gaining more attention in the field of biometrics. Here, we propose a lightweight CNN with batch normalization (BN). The main design focus was adding noise in the training process to prevent overfitting, as well as reducing internal covariance shift.

One framework for mapping convolutional neural networks on FPGAs was fpgaConvNet [12]. This approach allowed fast exploration of the design space by means of algebraic operations and it enabled the formulation of a CNN’s hardware mapping as an optimization problem. Different from the hardware conditions in this article, the FPGA core was an older process specification, and the core frequency and memory specifications used were lower than the hardware specifications in this article. The convolutional layer specification used was also relatively lower than the depth used in this article, and the BN proposed in this article was not used. Moreover, the characteristic specification of Sustained Performance would need to be further optimized and improved in the finger vein recognition system when compared with the method proposed in this article.

One design used an accelerator that trained the CNN using efficient frequency-domain computation [13]. It performed convolution using simple pointwise multiplications without the Fourier transforms by mapping all the parameters and operations into the frequency domain. The differences from the designs proposed in this paper were mainly due to the specification conditions of the hardware design being ASIC and FPGA platforms, the core frequency and integration of the precision control of the complex number computation, and the neural network architecture being relatively limited due to the design conditions. For the GPU calculation of the follow-up system, the design proposed in this paper was to run directly on the neural network IP of the FPGA to more completely calculate the results of finger vein recognition.

With the popularity of internet of things (IoT) systems, network security is the most important issue for people, and people generally do not want to upload too much personal information in security messages to the cloud server system. Therefore, “edge computing” has become an important research topic. Using this, we can build a security system that is not only safer but also faster because the transmission of information from the sensors is minimized.

Previous work on finger vein sensor devices can be categorized into two types: commercial and academic devices. Commercial devices are those developed by vendors, such as Hitachi, IDEMIA, and Mofiria. These sensors have been widely used in the market for several years. However, the manufacturers of these devices only provide the performance metrics of their products, including false acceptance rate (FAR) and false reject rate (FRR), without documenting their hardware implementation details. Additionally, the output image format of these devices is encrypted, so researchers are not able to obtain raw finger image data. To address this issue, we developed a finger vein capture device for academic use that can export captured vein images in a non-proprietary format. Furthermore, cost-effective components and simplicity were considered while developing our own device. A small, single-board computer (SBC) with NIR camera ware was used in our design, which makes it compact and feasible for various applications.

In this paper, we propose a low-complexity and lightweight CNN-based finger vein recognition system using edge computing, which can achieve a faster inference time and maintain a high-precision system recognition rate. Based on the specifications of the existing finger vein image acquisition products on the market, we designed and developed our own finger vein image capture device to collect the finger vein images for this study. Several preprocessing techniques and the modified CNN were combined to achieve better image quality and higher recognition accuracy. The low-complexity and lightweight CNN for the inference stage was designed based on an FPGA device that had limited resources, with only 13,300 logic slices, each with four six-input LUTs, eight flip-flops, and four clock management tiles, each with a phase and 220 DSP slices. Experiments were performed using a variety of application scenarios (including finger vein images obtained from fingers at different temperatures, heart rates (HRs), levels of cleanliness, etc.), illustrating the robustness and practicality of the proposed system.

The remainder of this paper is organized as follows. Section 2 describes the proposed system. Section 3 presents the experimental data. The system test results and comparisons are given in Section 4. Section 5 concludes this work.

## 2. Proposed System

**Figure 1:**
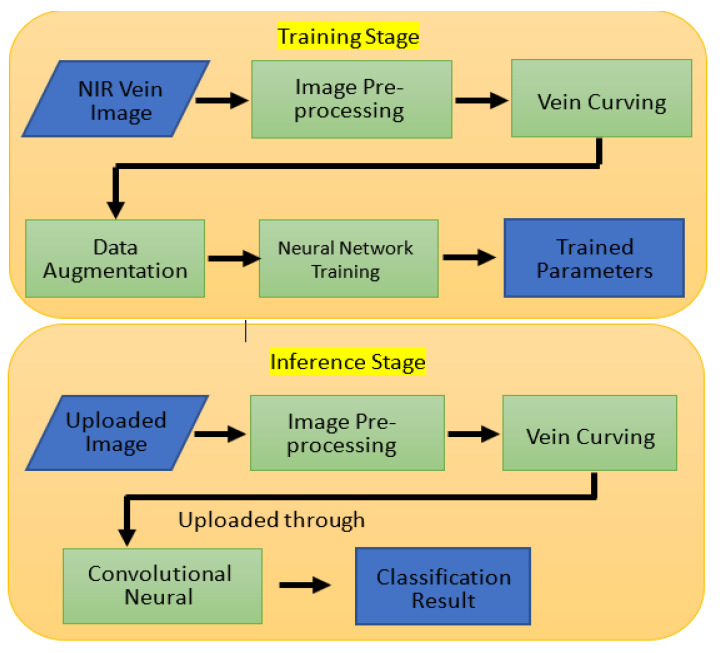
Proposed recognition system.

This study proposes a low-complexity and lightweight CNN-based finger vein recognition system using edge computing, which allows faster reasoning time to obtain the personal information identification results. Figure 1 shows the proposed recognition system, which includes the training stage and the inference stage. A detailed description is presented in the following sub-sections.

### 2.1. Training Stage

**Figure 2:**
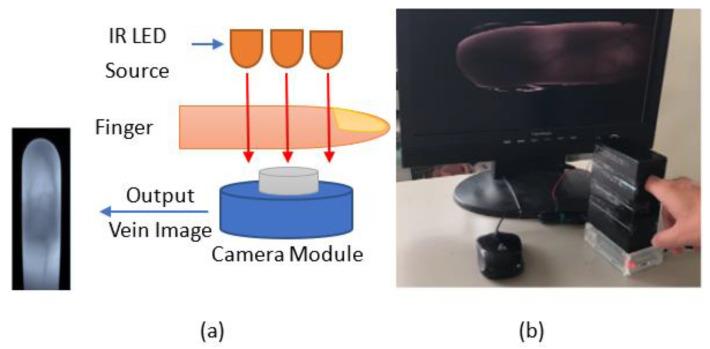
(**a**) Diagram of finger-vein collection system; (**b**) example of the operation of our device.

The original finger vein images were obtained from our capture device developed with the NIR 850 nm camera module shown in Figure 2. The image sensor was a Sony IMX219 in a fixed-focus module with an integral IR filter, and resolution was 8 M pixels with no infrared. This device was developed based on the specifications of existing commercial products. A simple system for illumination from the top side of the finger was designed. Therefore, to train the CNN on the finger vein features, we needed to perform several steps of preprocessing on the raw image. We conducted three main processes on the input data: vein curving, region of interest (ROI) capture, and scaling. The binary features and ROI of the finger vein images were calculated and used as the training data to obtain the parameters for the modified CNN, which were then used in the inference stage.

**Figure 3:**
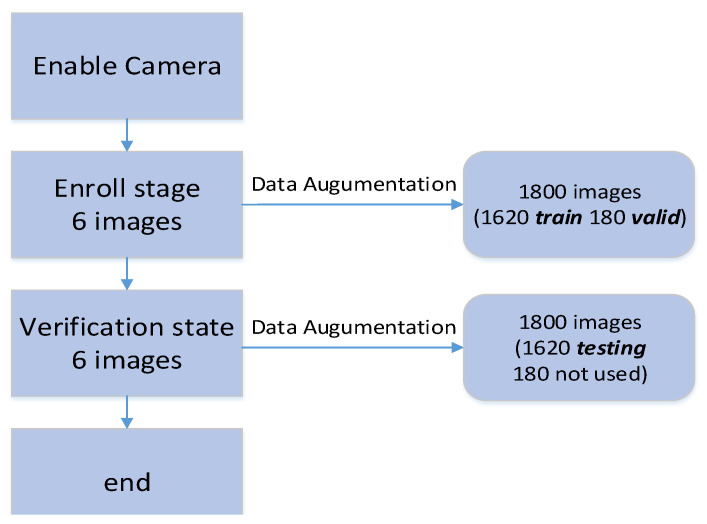
Finger vein dataset collection flow.

In order to collect the training and testing datasets, the following steps were used, as shown in Figure 3. First, the finger vein images of 10 people were acquired through the camera equipment. Six real-time finger vein images were taken for each person, yielding a total of 60 original finger vein image files of the 10 people. After data augmentation, the total number was 1800. The images were then divided into two categories: 1620 images for training and 180 images for validation. In addition, the real-time finger vein images for these 10 people were re-acquired, and then another 60 original finger vein image files were obtained. After data augmentation, a total of 1800 images were obtained. From these, 1620 images were randomly selected for the system testing.

(1) Image Preprocessing.

In the actual application of the finger vein recognition device, it was necessary to consider various factors that can cause misjudgments when capturing the current finger vein image. For example, if the size of the finger is different, or the image is too large, the feature list is required, and if the image is too small, invalid edges where the edges of the fingers are overexposed cannot be removed. The depth of the fingerprint on the finger surface is also an important item that needs to be removed from noise interference. Thus, one must determine how to prevent the noise image of fingerprints or skin surface wear-and-tear from overlapping with the vein image. The surface temperature of the finger, affected by situational factors in various climates, may cause slight changes in the size of the pattern, and the training conditions must be added when the training dataset is initially established.

With reference to the structure of the standard finger vein devices, such as the Hitachi H1, the external light overlaps with the NIR light. The sensitivity and denoising ability of the CMOS lens react to the CMOS lens. The most common problems are irregular finger image edges and light overlap exposure. Therefore, we needed to perform image preprocessing for each angle and noise simulation or image after adding conditions when creating the initial finger vein image for each person being tested.

The main purpose of this stage was to generate the corresponding input pattern for the training network. First, we converted the captured RGB image into grayscale. Then, we scaled the image pixels ranging from 0 to 1. This normalization operation was to prevent a large gradient during training.

(1) $$X_{norm}=\frac{X-X_{min}}{X_{max}-X_{min}}\;,for\;i=1\;\ldots N\;$$

**Figure 4:**
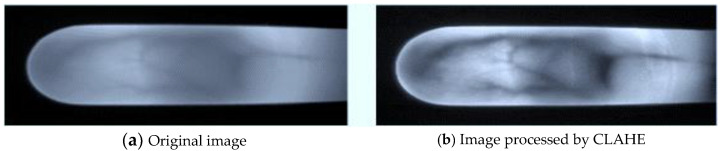
Finger vein image processed by CLAHE.

After normalization, we enhanced the normalized image in order to emphasize the vein features. We implemented the contrast-limited adaptive histogram equalization (CLAHE) method, as shown in Figure 4. Compared with global histogram equalization, CLAHE amplifies local features by dividing images into small blocks called “tiles.” Then, each tile equalizes the histogram as in global equalization.

**Figure 5:**
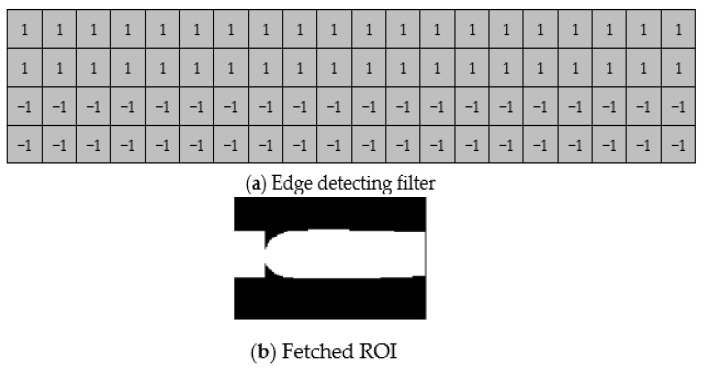
(**a**) Edge detection filter used for ROI segmentation; (**b**) ROI result.

Since only a portion of the image has the features of finger veins, we needed to fetch the ROI of the image, as shown in Figure 5. First, we used an edge detection filter with 20 × 4 pixels, which was divided into upper and lower parts. The upper part was filled with 1, and the lower part was filled with −1. By filtering the finger image, the upper part of the image produced a maximum value, while the lower part produced a minimum value.

**Figure 6:**
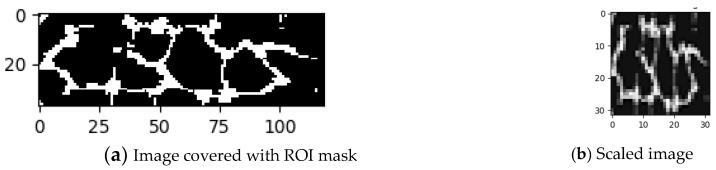
(**a**) Finger vein ROI result; (**b**) resized image for training.

For the sake of hardware structure and resource utilization, we scaled both our training and testing datasets to 32 × 32 pixels after we fetched the ROI of the finger vein, as shown in Figure 6.

(2) Data Augmentation

A neural network usually needs a large amount of data to learn the differences between each category. Here, we performed the augmentation process considering the following:

(1) Since we used a high-resolution camera and scaled to a smaller size to perform the inference, the quality of the source image we captured was stable. There was no need for a training network with Gaussian noise, coarse dropout, or random brightness.
(2) We did not restrict the user’s finger position, so the finger could shift or rotate slightly. To address this issue, our augmentation process mainly focused on shifting and rotating.

**Figure 7:**
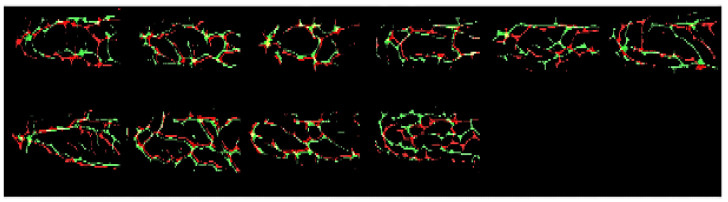
Difference between “registration” and “verification” datasets.

**Figure 8:**
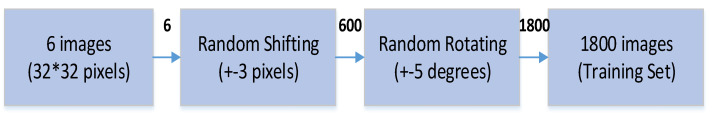
Data augmentation strategies.

In the data augmentation phase, we compared the “registration” data, shown as green lines in Figure 7, and the “verification” data, shown as red lines. Since the tolerance of rotation error mostly varied by plus–minus five degrees and the error of shifting varied by plus–minus three pixels, we only augmented the dataset with random shifting and random rotation. The two different datasets and the augmentation strategy are shown in Figure 7 and Figure 8. After the dataset was completed, there were 1620 images for training, 180 for validation, and another 1620 for testing.

(3) Neural Network Training

For neural network training, we used convolutional architecture for fast feature embedding (Caffe) to establish the architecture and obtain trained parameters for the purpose of FPGA inference. Caffe is a deep learning framework designed for expression, speed, and modularity [14]. In practice, a complete Caffe training consists of several classes: Blob, Layer, Net, and Solver. Blob is an N-dimensional array-storing data type defined in Caffe; its main objective is to hold data, derivatives, and parameters in training and inference dataflow. Layer is Caffe’s fundamental unit of computation; basic layers such as Convolution and Pooling are defined in this class.

First, users can customize their own neural network using several layer declarations and then establish the network structure file, namely, the prototxt file. Second, in order to train with our own dataset, image data had to be transformed into LMDB format and thus referenced by the prototxt structure. Compared with TensorFlow [15], Caffe has a faster training speed and lower memory requirements.

### 2.2. Inference Stage

**Figure 9:**
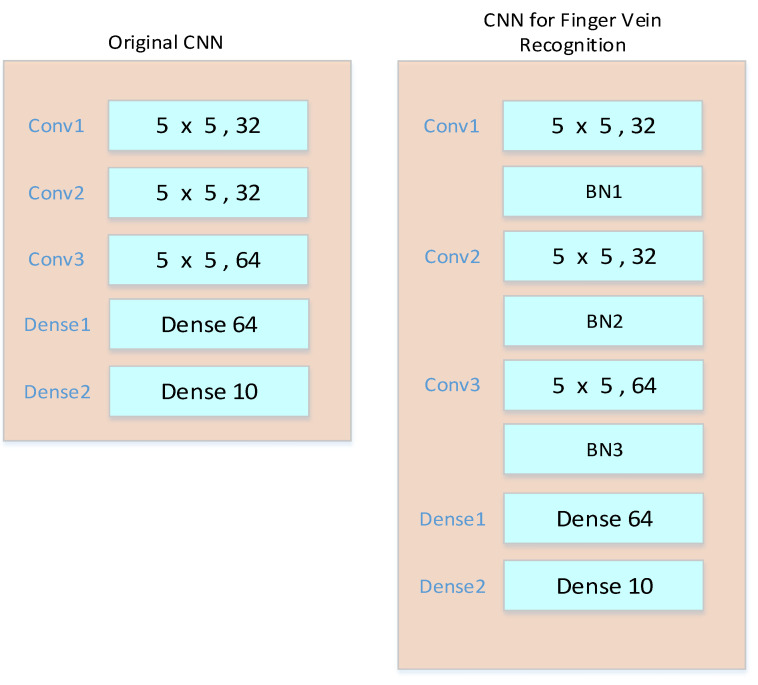
Original and modified CNNs used for vein recognition.

A modified CNN for finger vein recognition is proposed in this paper. The original CNN was obtained from the MNIST training model provided by Caffe, and the modified CNN with batch normalization (BN) can obtain higher accuracy. The original and modified CNNs are shown in Figure 9.

Vivado HLS was used to accelerate our neural network. The modified CNN consisted of three convolutional layers, with BN after each layer. This intellectual property (IP) featured im2 col convolution, which can be supported by the matrix multiplication DSP provided by Xilinx. The acceleration ratio was 120× compared with the network, running in a Linux environment with a 650 Mhz ARM cortex-A9 dual core CPU. Since the preprocessing in the inference phase was similar to the preprocessing in the training phase, the following sections focus on our neural network IP design. First, we designed and modified the complexity and light weight of the CNN. In order to meet the requirement of FPGA hardware system computing efficiency, we had to ensure that the convolution algorithm occupied the largest proportion of the entire computing resources and that data were reused in the convolution stage. We implemented general matrix multiplications (GEMMs) to optimize the convolution operation in the IP design. GEMMs basically expand the feature map and the kernel into two main matrices and then complete the convolution by multiplying the two matrices. After the two matrices are completed, the multiplication is performed, based on a 5 × 5 feature map with two channels and a 3 × 3 kernel. Maximum pooling is carried out by selecting the maximum value within the pooling size. The fully connected layer can be regarded as a 1 × 1 convolution. After the convolutional layer chain, the feature map is flattened into a one-dimensional (1D) stream and convolved with the input fully connected parameters. Finally, we used Jupyter Notebook [16] for connecting the uploaded image and the IP. Jupyter Notebook is an interactive computing environment that enables users to manipulate their input data and calling IP directly. The detailed design is described as follows.

(1) IP Structure

**Figure 10:**
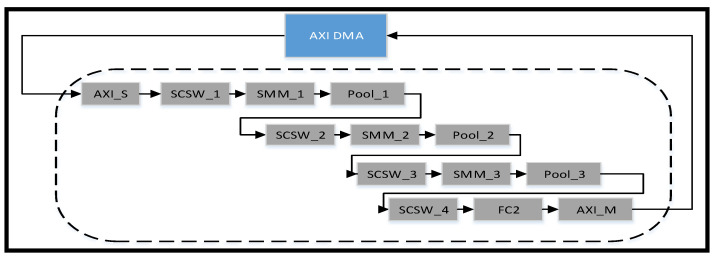
Convolution IP dataflow. AXI Stream (AXI_S), Stream Convolution Sliding Window (SCSW), Stream Matrix Multiplication (SMM), Fully Connected (FC) layer.

GEMMs [14,17], Winograd Transform [18], and Fast Fourier Transform [13] are well-known solutions to the issue of ensuring that the convolution algorithm occupies the largest proportion of the entire computing resources and that data are reused in the convolution stage. We implemented GEMMs to optimize the convolution operation in the IP design. The overall convolution IP flow is illustrated in Figure 10.

(2) GEMMs

**Figure 11:**
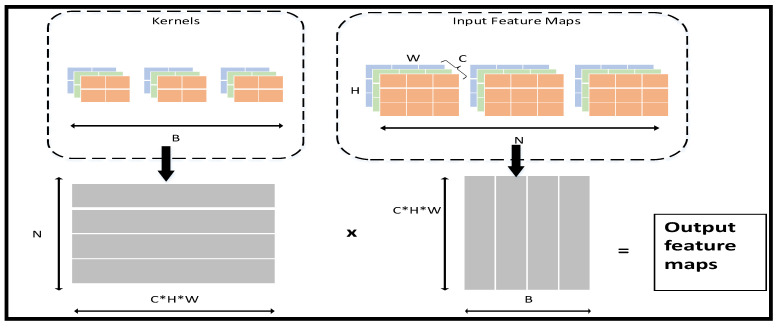
GEMMs’ convolution operations.

Before stepping into the dataflow in the IP, we need to introduce GEMMs. In a CPU or GPU, a common way to process convolutional and fully connected layers is to map them as matrix multiplication. GEMMs basically expand the feature maps and the kernels into two main matrices, and then the convolution is performed by multiplying the two matrices by each other. The details are illustrated in Figure 11.

(3) SMM

**Figure 12:**
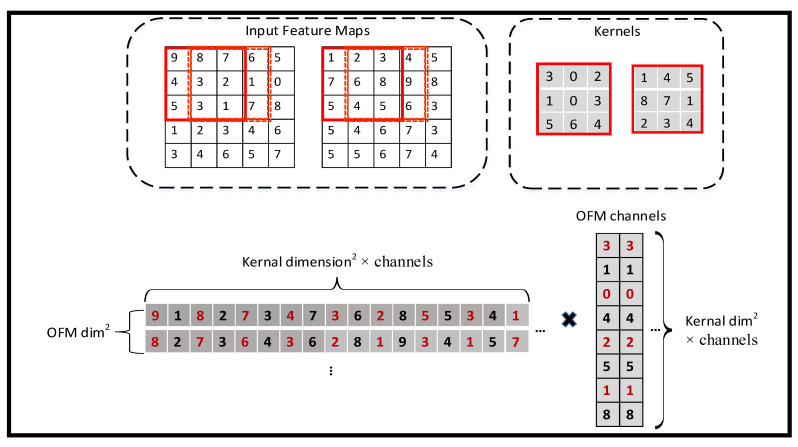
Convolution consisting of SCSW and SMM.

After the two matrices are finished, the multiplication is carried out in this stage. Take a 5 × 5 feature map and 3 × 3 kernel with two channels as an example. Figure 12 shows the calculated details for this simple example.

Max pooling is performed by choosing the maximum value within the pooling size. To achieve this goal, we compared each input value with the previous value. That is, three comparisons were carried out for a 2 × 2 max pooling.

Technically, a fully connected layer can be viewed as a 1 × 1 convolution. After the convolutional layer chains, the feature maps are flattened into a 1D stream and convolved with the input fully connected parameters.

(4) Batch Normalization (BN)

Batch normalization (BN) [19] is known for reducing the internal covariate shift by normalizing the inputs to have a mean of 0 and a standard deviation of 1. Two parameters, β and γ, are obtained from mini-batches in the training phase and used for the inputs in the inference stage. γ is the scale of the standard deviation parameter, and β is the shift of the mean parameter. A detailed formula for the BN during the training stage is shown in Equation (2), where B is the mini-batch size, C is the depth of input feature maps (IFMs), N is the depth of output feature maps (OFMs), OF_W_ is output feature map width, OF_H_ is output feature map height, σ is mini-batch variance, and ε is a negligible constant.

(2) $$Y^{\mathrm{BN}}\left[B,N,{\;\mathrm{OF}}_{W},{\mathrm{OF}}_{H}\right]=\frac{\left(X^{\mathrm{BN}}\left[B\;,C\;,{\mathrm{OF}}_{W},{\mathrm{OF}}_{H}\right]\right)}{\sqrt{\sigma^{2}+\epsilon }}\gamma +\beta \;$$

At the inference stage, β and γ are used for scaling and shifting after the inputs are normalized by the maximum and minimum values in the input feature map. The scaling and shifting parts only involve multiplication, while normalization requires division, which could consume a large amount of computational resources on our FPGA board. The normalization formula can be described as follows:

(3) $$X_{norm}=\frac{X-X_{min}}{X_{max}-X_{min}}\;,for\;i=1\;\ldots N.\;$$

Now, we have two ways of implementing this equation:

(1) Directly compute the division in the IP. (2) Preprocess the parameters by Python before sending them to the data stream. Initially, we tried the first approach, but this failed because our convolution parameters ranged from 0 to 1 while the batch norm parameters pretrained by Caffe exceeded this range. Fixed-point arithmetic will overflow in this case. Therefore, we moved to the second approach, because the output was obtained by the input divided by a variable pretrained by the neural network. To simplify the hardware architecture and save resources, we divided the batch norm layer parameters using Numpy before sending them into the data stream.

## 3. Experimental Data

The finger vein datasets used for training and testing were as follows.

### 3.1. Laboratory’s Own Dataset

**Figure 13:**
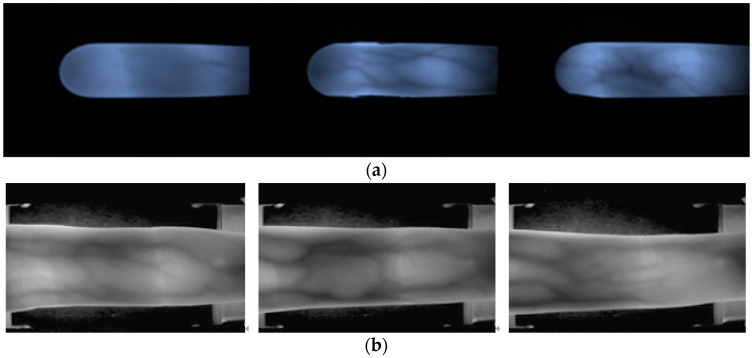
(**a**) Laboratory dataset and (**b**) SDUMLA-HMT database.

Using our self-developed equipment, we randomly selected 10 people and obtained their index finger images six times to create a dataset containing a total of 60 images with a resolution of 320 × 240. After performing data enhancement on these 60 images, a total of 1800 images were generated, including 1620 images as the training dataset and 180 images as the validation dataset. In addition, 6 images of the finger veins of the same 10 people were captured again to yield a total of 60 images. After that, these 60 images were augmented to produce a total of 1800 images, from which 1620 images were randomly selected as the system test dataset. Figure 13a shows some examples of the finger vein images from our own dataset. In practical applications, the same tester will produce different samples of finger vein images at different times.

### 3.2. SDUMLA-HMT Finger Vein Dataset [20]

The SDUMLA-HMT finger vein dataset consisted of 106 people, and for each person, the index fingers, middle fingers, and ring fingers of both hands had been captured. The collection of each of the six fingers had been repeated six times, and the resolution of each image was 320 × 240 pixels. From among all 3816 images, we selected 10 people and 60 finger images for experimentation. Figure 13b shows some examples from the SDUMLA-HMT database. We built our own dataset based on the paradigm of the SDUMLA-HMT dataset. Each tester will have a variety of similar but different vein images.

### 3.3. Temperature Factor Test Image

**Figure 14:**
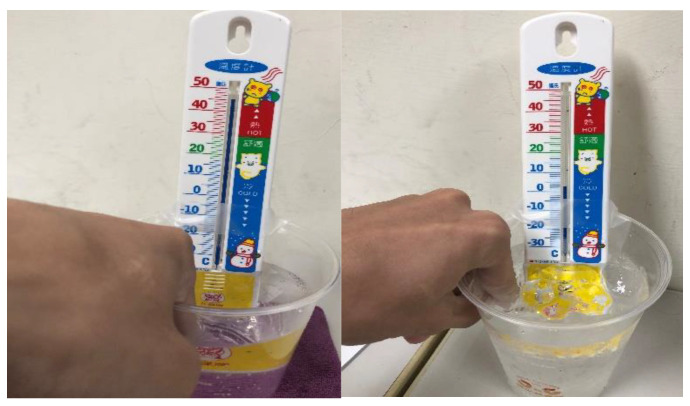
Showing 2 °C and 45 °C finger temperature testing.

**Figure 15:**
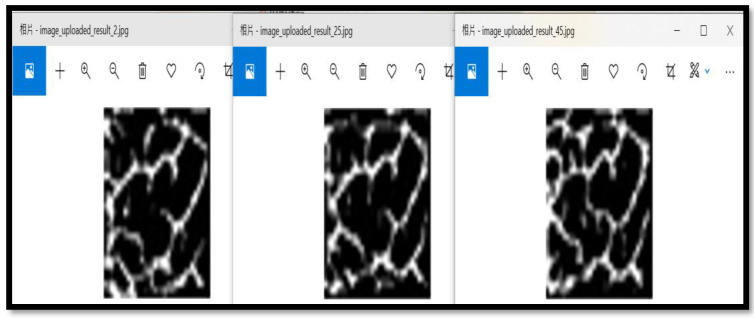
Showing 2 °C, 25 °C, and 45 °C finger vein diagrams with different surface temperatures.

This experiment was specifically aimed at assessing finger vein image sampling results under different temperature conditions. Three cold and hot temperature values were used (2 °C, 25 °C, and 45 °C,), which may be caused by the external temperature of the human body. Figure 14 shows the real test scenario in which a finger was immersed for about 30 s in a low temperature of 2 °C (left figure) and a high temperature of 45 °C (right figure). Figure 15 shows finger vein images after the three temperature measurements at a low temperature of 2 °C (left figure), a room temperature of 25 °C (middle figure), and a high temperature of 45 °C (right figure).

### 3.4. Sampling of Dirty Finger Surfaces

**Figure 16:**
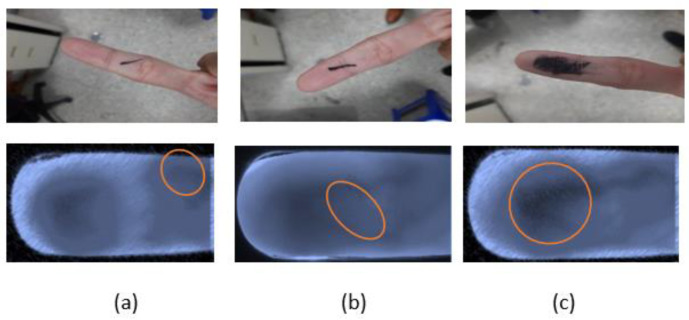
(**a**) Slightly dirty; (**b**) cut marks; (**c**) simulated birthmark.

This experiment was specifically aimed at assessing the capture and testing of images that might be affected by having something on the surface of the finger (dirt, birthmarks, cut marks, etc.). The purpose of this experiment is to increase the complexity of the test phase and show the robustness of our approach. As shown in Figure 16, we considered several interference features that are most likely to cause skin surface damage, and collected the finger vein images with a slightly dirty skin surface, cut marks, and simulated birthmarks.

### 3.5. HR and Blood Pressure

**Figure 17:**
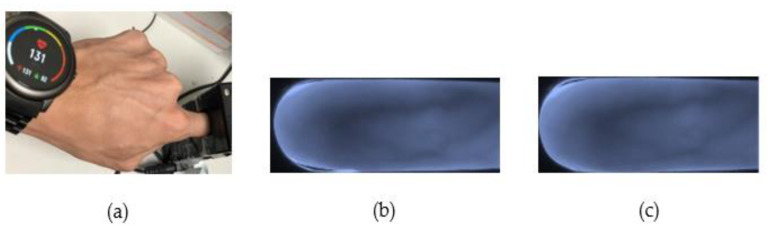
HR sampling: (**a**) HR is 130/min; (**b**) before exercise; (**c**) after exercise.

This experiment was specifically aimed at assessing the effect of differences in HR or blood pressure, e.g., in people suffering from disease or after exercise, confirming that this is an experimental design for the weak elasticity test of the basic common sense in venous blood vessels. As shown in the sampling diagram in Figure 17, we used a heart rhythm test to capture heart rhythm data after 30 min of exercise, and then checked the accuracy of the finger vein recognition by our approach. The test subjects are young and healthy without disease. Cases of other conditions, such as any pathological or physiological condition, are not included in the testing considerations.

## 4. Experimental Results

### 4.1. Laboratory’s Own Dataset

#### 4.1.1. Impact of Input Image Size and Enhancement

**Figure 18:**
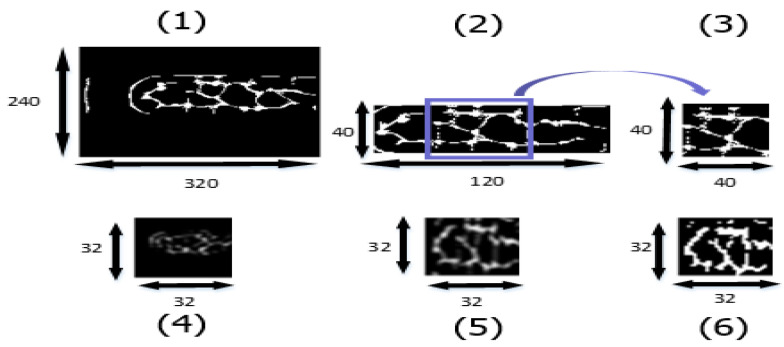
Six different training and testing datasets.

Examples of the datasets used in this experiment are shown in Figure 18. We implemented six different strategies to make the training and testing datasets: (1) whole image using original resolution, (2) fetching smaller 120 × 40 ROI with no expression, (3) fetching 40 × 40 of the central finger area with no expression, (4) whole image downsized to 32 × 32, (5) fetching smaller 120 × 40 ROI with expression to 32 × 32, and (6) fetching smaller 120 × 40 ROI with expression to 32 × 32 and binary.

Table 1 indicates that Dataset (6) had the best accuracy among our datasets. The reason was that Dataset (6) not only preserved the whole finger vein features but also emphasized downsized features with binarization.

**Figure 19:**
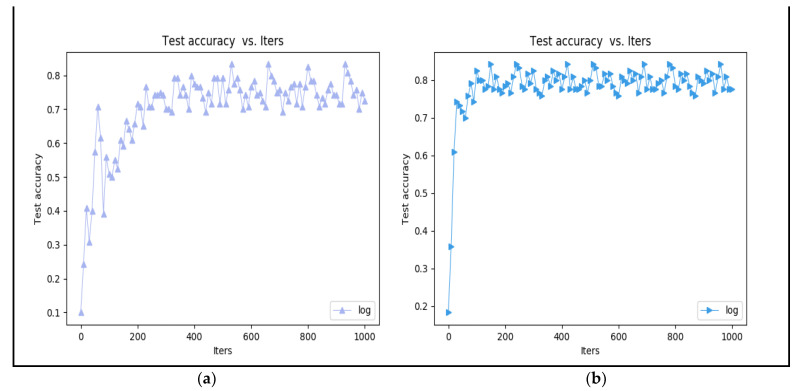
Training (**a**) without CLAHE and (**b**) with CLAHE.

In addition, we compared the accuracy of the datasets with and without CLAHE enhancement. As Figure 19 shows, we found that with CLAHE it can quickly converge in iteration and this increases the accuracy rate; on the other hand, if the CLAHE method is not used, the iteration of training must spend more time stabilizing the convergence and this affects the accuracy rate. The training accuracy increased by about 5% after implementing CLAHE.

#### 4.1.2. Impact of BN

BN is a good way to increase both the training speed and the model accuracy when adding noise to the data batch. We utilized our modified CNN to verify the effect of BN.

**Figure 20:**
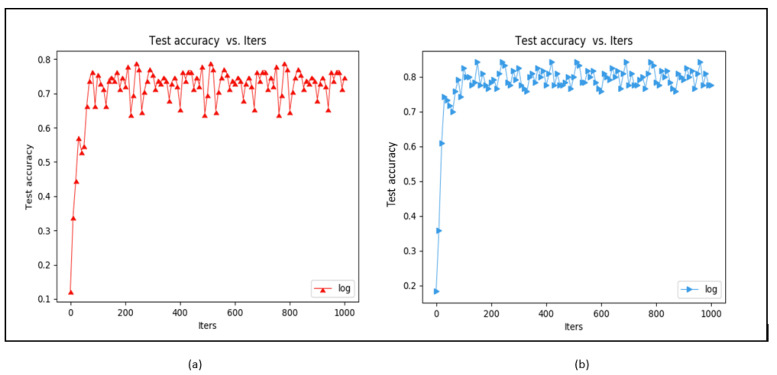
Training (**a**) without BN and (**b**) with BN.

As Figure 20 shows, we found that with BN it can quickly converge in iteration and this increases the accuracy rate; on the other hand, if the BN method is not used, the iteration of training must spend more time stabilizing the convergence and this affects the accuracy rate. With BN, the finger vein training accuracy was increased by about 8–10% and the accuracy converged faster. Given these two results, we concluded that BN benefited our training model.

#### 4.1.3. Impact of Optimizers

Different optimization strategies may lead to different training results. Here, we tested five methods to train our dataset. The parameters were set to default and they were run for 1000 iterations.

Among these optimizers, Adam performed the best for our finger vein dataset, as shown in Table 2. Since Adam combines the RMSProp with momentum, the first order and second order of the gradient were considered in the process of weight updating. Not only does the gradient converge to the curve faster, but the amplitude of the curve is also smaller when implementing Adam.

#### 4.1.4. Impact of FPGA Acceleration

**Figure 21:**
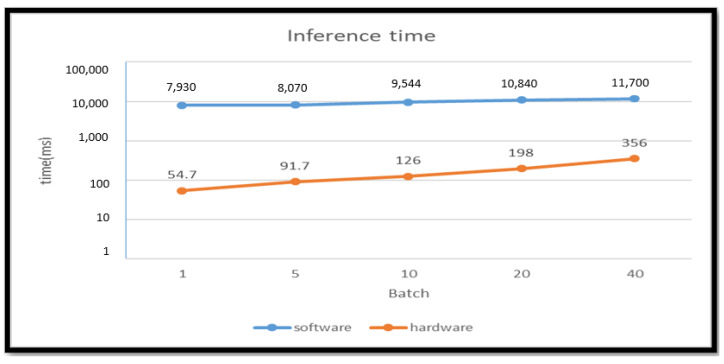
Inference time comparison.

We used the CPU embedded on the board as the reference for the software approach. On the software side, Theano was used to build the modified CNN framework. Using PYNQ (Python productivity for Zynq), the FPGA ran in the Python environment. Theano compiles the network framework directly in Python, while Caffe compiles it with C++, so significant resources are wasted by using Caffe. Figure 21 shows the inference time difference between the two approaches. We define the way to use the modified CNN framework to assess inference cost time against hardware time and used the Caffe compiles framework to assess inference cost time against software. The inference time of convolution can be accelerated from 7.93 s to 54.7 ms for recognizing a single image.

#### 4.1.5. Temperature Factor Testing Results

**Figure 22:**
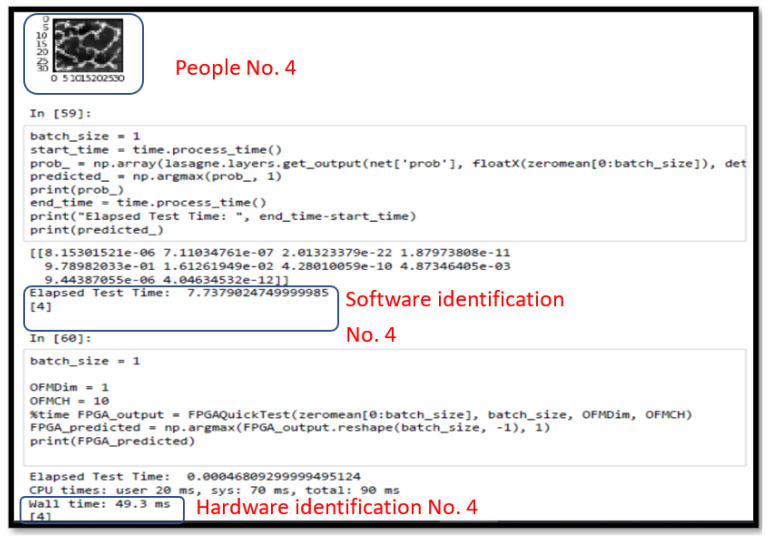
Recognition result with finger surface temperature of 2 °C.

In this experiment, Participant No. 4 was used as the test sample. He put his finger in ice water (2 °C), stood still at room temperature (25 °C), and then immersed the finger in hot water (45 °C). The temperature status of each finger was captured in sequence. After the vein image was taken, it was input into the system of this paper for body identification. Figure 22 shows the result of the ice water test at 2 °C. First, the finger vein image of Participant No. 4 was read. Then, the software computing system took about 7.74 s to determine that it was Participant No. 4. Finally, the FPGA hardware acceleration system took about 48.3 ms to determine that it was Participant No. 4. The hardware was more than 150 times faster at calculating the results than the software. The simultaneous synchronization was aimed at comparing the results at 25 °C, 45 °C, and 2 °C, and it proved that the finger surface temperature did not affect the finger vein recognition results. This greatly improves the accuracy of the system for use in a variety of countries and regions with different ambient temperatures. It also demonstrates the high recognition rate and accuracy.

#### 4.1.6. Dirty Finger Surface Testing Results

According to the experimental design presented in Part 4 of Section 3, we aimed at assessing the potential interference of conditions that may occur on the surface of the finger, including dirt, cut marks, and birthmarks. The test results correctly identified the participant, and as the results were the same as those in the previous paragraph, they are not repeated here. These experiments proved that this research proposes a low-complexity and lightweight CNN-based finger vein recognition system with edge computing that uses faster hardware to reduce the inference time to obtain high-accuracy security information recognition results.

#### 4.1.7. HR and Blood Pressure Testing Results

According to the experimental design presented in Part 5 of Section 3, we aimed at assessing the impact of different physiological conditions in people, specifically blood pressure changes caused by disease factors or heart rhythm variability caused by exercise. We found that the tester’s finger veins after the simulation state were affected, but after inputting this into the system for identification, the test results correctly identified the participant.

### 4.2. SDUMLA-HMT Database

**Figure 23:**
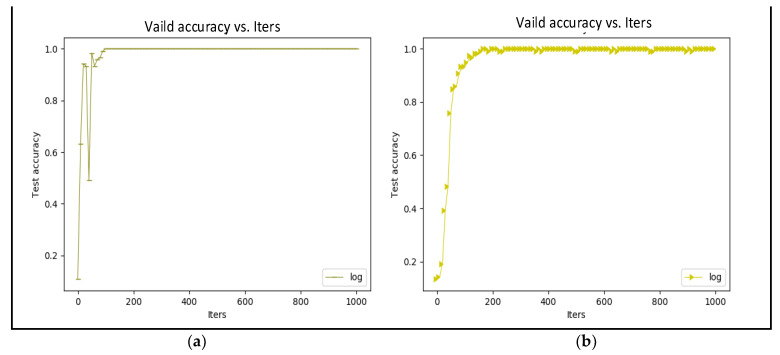
SDUMLA-HMT validation accuracy (**a**) trained with BN and (**b**) trained without BN.

In our own dataset, there were 6 original images for each person, making 1620 images for training after data augmentation, and then another 6 images were captured for testing. In the SDUMLA-HMT database, only six original images were captured for each finger. There were no other source images for testing. Thus, we only tested the training accuracy using the validation dataset to verify the feasibility of our proposed finger recognition CNN model. Figure 23 illustrates that both types of training accuracy converge after 100–200 iterations. However, training with BN converges faster than training without it. BN not only reduces the internal covariance shift but also adds noise to make the training process faster.

### 4.3. Throughput Estimation

The throughput provided by the hardware can be estimated by the following formula:

(4) $$Throughput=\sum \left(Multiply\;Accumulate\;\left(MAC\right)\;in\;one\;clock\;\times clock\;frequency\right).$$

Figure 21 indicates that we could inference 18.21 images in 1 s, and the workload of our model was 0.0248 GOPS. Hence, the actual performance is calculated as 0.451 GOPS.

### 4.4. Comparison Results

Table 3 shows the results of a comparison with other works [6,7,10,11], which again verifies that the embedded system proposed in this paper can achieve quite high accuracy. For example, for a well-designed Gabor filter bank proposed by Ref. [6], which can enhance the original gray image and extract more accurate finger vein texture features, the verification was still based on the use of MATLAB software in a PC hardware device environment. The verification results showed that the design of the finger meridian image acquisition device based on near-infrared spectroscopy was very important to obtain high-precision finger vein recognition results. In comparison, based on low-cost, low-complexity, and lightweight FPGA hardware capabilities, our paper fully considered the preprocessing of the acquisition and the influence of finger veins as the direction of product design. Both systems use their own designed device to build the database, but with a difference of platform, either PC or FPGA.

In Ref. [7], the software method was based on a PC hardware interface and the use of MATLAB software, and was especially focused on the edge fitting item of the segmentation model, where the active contour occurs when the finger vein image is captured, and accurately completes the finger vein image segmentation. In comparison, for the hardware accelerated computing method proposed in our paper, the fixed LED light source was based on an NIR camera and was applied to simplify the CNN. On the FPGA, it can also obtain high-accuracy real-time identification of finger vein images. Both systems use their own designed device to build the database, with a difference being again the platform, PC or FPGA.

In Ref. [10], the paper proposed a lightweight deep-network finger vein recognition algorithm which can effectively extract and match the features of finger vein images and has high recognition accuracy and matching speed. The software method was based on a PC hardware interface and worked on a graphics processing unit (GPU) device for training and inference. The database used SDUMLA-HMT and PKU-FVD [10]. Compared with the hardware accelerated computing method proposed in this paper, that lightweight deep-network finger vein recognition algorithm needed a high-performance GPU, while our paper has proposed a general, simplified CNN model that could work on an FPGA platform, not needing a GPU, and we also used our own design device to build the database and verified it with SDUMLA-HMT.

In Ref. [11], the authors proposed a CNN-based embedded identification system which used a multi-intensity illumination strategy to improve image quality, and they proposed using their FVRAS-Net to extract features for both the recognition and anti-spoofing tasks and thus to guarantee real-time performance of the embedded system. The software method was based on a PC hardware interface and used a graphics processing unit (GPU) device for training and a platform of the NVIDIA development board Jetson TK1 for inference. The database used IDIAP, USM, SDUMLA-HMT, MMCBN, and SCUT-SFV [11], and they also built their own database using a commercial finger vein biometric system. In comparison, our paper has proposed a general, simplified CNN model that could work on an FPGA platform, not needing a GPU and high performance in the embedded system. For the algorithm, not only was our CNN model simpler than their FVRAS-Net, but the hardware was easier and lower in cost for edge computing. Our system used our own designed device to build the database and then verified with SDUMLA-HMT.

## 5. Conclusions

In this paper, we proposed a low-complexity and lightweight CNN-based finger vein recognition system with edge computing that can achieve a faster inference time and maintain a high-precision system recognition rate. Based on the equipment specifications of the existing finger vein image acquisition products on the market, we designed and developed our own finger vein image capture device to collect the finger vein images for this study. Specifically, a set of darkroom photography equipment that conforms to the NIR wavelength of 850 nm was used.

The modified CNN-based finger vein recognition system consists of three convolutional layers, and BN is performed after each layer. Several preprocessing techniques and the improved CNN were combined to obtain better image quality and higher recognition accuracy. The implementation details were described in this article. At the same time, experiments were performed using a variety of application scenarios (including finger vein images obtained from fingers at different temperatures, HRs, levels of cleanliness, etc., as well as standardized personal finger vein images). These experiments illustrated the robustness and practicality of the proposed system.

The proposed system has potential for both design flexibility and commercialization. The market application goal was to conform to the structure and market of miniaturized community security system applications. For a community-based smart security system connected to a smart city, although it would be necessary to add a very small amount of personal recognition data to the database, we would only need to retrain the personal feature items to complete the security system update and maintain high-efficiency and high-accuracy functions.

## Figures and Tables

**Table 1:** Training accuracy summary of six different datasets.

Dataset	Training Accuracy (by Choosing Highest in Last 100 Iters)
[1]	91.33%
[2]	94.16%
[3]	93.33%
[4]	85.00%
[5]	92.77%
[6]	95.83%

**Table 2:** Comparison of different optimizers (unen is unenhanced dataset and en is enhanced dataset through CLAHE).

	Method	Without BN (unen/en)	With BN (unen/en)
Optimizers			
SGD		8%/10%	75%/74%
RMSProp		38%/54%	50%/65%
Nesterov		56%/68%	70%/72%
Adam		50%/74%	70%/75%
AdaDelta		12%/7%	17%/32%
AdaGrad		55%/70%	81%/72%
RMSProp		38%/54%	50%/65%

**Table 3:** Comparison of results with other works.

	Method	Ref. [6]	Ref. [7]	Ref. [10]	Ref. [11]	Proposed
Item						
Algorithm		Illuminance control	Kernel fuzzy C-means.	Self-define a lightweight convolutional model	FVRAS-Net	Simplify CNN model
Algorithm complexity		High, verification with MATLAB	Medium, verification with MATLAB	High, verification with GPU	Medium, verification with Embedded-Board	Low, verification with FPGA
Finger vein identification device		Their own design device	Their own design device	No, only public database	Commercial finger-vein biometric system	Our own design device
Platform		PC-Based	PC-Based i5-6500 CPU (3.2 GHz) and 8-GB RAM	PC-Based I7-8700 CPU (3.2 GHz) and Nvidia 3090 Ti (24-GB video memory) GPU	Embedded-Board NVIDIA development board Jetson TK1, Nvidia 1080 Ti GPU	FPGA Xilinx XC7Z020 650 MHz on ZYNQ and 512 MB RAM
Database		Their self-built database	Their self-built database	SDUMLA-HMT and PKU-FVD	Their self-built database, IDIAP, USM, SDUMLA-HMT, MMCBN and SCUT-SFV	Our self-built database and SDUMLA-HMT
Recognition accuracy rate (%)		95.15%	98.27%	99.3%	97.82%	95.82%
EER (%)		4.85%	2.35%	--	2.18%	4.17%
Inference time (s)		2.96 s	--	14.2 ms	13.11 ms	0.356 s

## Data Availability

Not applicable.
